# Estrogen-Dependent Dynamic Profile of eNOS-DNA Associations in Prostate Cancer

**DOI:** 10.1371/journal.pone.0062522

**Published:** 2013-05-03

**Authors:** Simona Nanni, Aurora Aiello, Agnese Re, Alessandro Guffanti, Valentina Benvenuti, Claudia Colussi, Luis Jaime Castro-Vega, Armando Felsani, Arturo Londono-Vallejo, Maurizio C. Capogrossi, Silvia Bacchetti, Carlo Gaetano, Alfredo Pontecorvi, Antonella Farsetti

**Affiliations:** 1 Department of Experimental Oncology, National Cancer Institute Regina Elena, Rome, Italy; 2 Medical Pathology Institute, Catholic University, Rome, Italy; 3 Institute of Cell Biology and Neurobiology, National Research Council (CNR), Rome, Italy; 4 University of Messina, Messina, Italy; 5 Genomnia srl, Lainate, Milan, Italy; 6 Institut Curie, Paris, France; 7 Istituto Dermopatico dell’Immacolata, Rome, Italy; 8 Goethe University, Frankfurt, Germany; University of Bristol, United Kingdom

## Abstract

In previous work we have documented the nuclear translocation of endothelial NOS (eNOS) and its participation in combinatorial complexes with Estrogen Receptor Beta (ERβ) and Hypoxia Inducible Factors (HIFs) that determine localized chromatin remodeling in response to estrogen (E2) and hypoxia stimuli, resulting in transcriptional regulation of genes associated with adverse prognosis in prostate cancer (PCa). To explore the role of nuclear eNOS in the acquisition of aggressive phenotype in PCa, we performed ChIP-Sequencing on chromatin-associated eNOS from cells from a primary tumor with poor outcome and from metastatic LNCaP cells. We found that: 1. the eNOS-bound regions (peaks) are widely distributed across the genome encompassing multiple transcription factors binding sites, including Estrogen Response Elements. 2. E2 increased the number of peaks, indicating hormone-dependent eNOS re-localization. 3. Peak distribution was similar with/without E2 with ≈ 55% of them in extragenic DNA regions and an intriguing involvement of the 5′ domain of several miRs deregulated in PCa. Numerous potentially novel eNOS-targeted genes have been identified suggesting that eNOS participates in the regulation of large gene sets. The parallel finding of downregulation of a cluster of miRs, including miR-34a, in PCa cells associated with poor outcome led us to unveil a molecular link between eNOS and SIRT1, an epigenetic regulator of aging and tumorigenicity, negatively regulated by miR-34a and in turn activating eNOS. E2 potentiates miR-34a downregulation thus enhancing SIRT1 expression, depicting a novel eNOS/SIRT1 interplay fine-tuned by E2-activated ER signaling, and suggesting that eNOS may play an important role in aggressive PCa.

## Introduction

Nitric oxide (NO) and its synthases attained celebrity among oncologists because of the evidence of frequent deregulation of NO production in several tumors, including prostate cancer (PCa, [1], [2], [3], and of the discovery of a key role played by the endothelial NOS (eNOS) in tumor maintenance and progression [1], [3], [4]. Our prior experimental results have provided demonstration of the physiopathological role of eNOS in three cellular contexts: normal human endothelial cells (HUVEC) before and after treatment with 17β-estradiol (E2); epithelial cell cultures from PCa explants grown in basal condition or with E2; and prostate tissue specimens from PCa patients. Confocal microscopy and immunohistochemistry have documented, in particular, eNOS nuclear translocation in all three experimental models [1], [5] and provided the following evidence: (i) eNOS-NO ‘nuclear’ signaling is a key pathway in endothelial cell response to angiogenic stimuli and in the acquisition of a more aggressive phenotype in PCa; and (ii) the existence and functional role of crucial combinatorial complexes on chromatin, eNOS/ERα specifically involved in the maintenance of vascular homeostasis [6], [7] and eNOS/ERβ, eNOS/HIF-1α or eNOS/HIF-2α specifically associated to adverse clinical outcome of PCa [1]. In the tumor model, these complexes determine localized remodeling of the chromatin in response to estrogen and hypoxia stimuli, resulting in transcriptional regulation of prognostic target genes [1]. Whether eNOS and its partners are present as a constellation of coordinate complexes or in the form of a macro-multifactorial complex remains to be evaluated.

In recent years, a relevant role in human cancer initiation, progression and metastasis has been assigned also to dysregulation of microRNAs (miRs) [8], [9]. How the expression of prognostic target genes is regulated in the context of PCa is currently under investigation although several reports [10], [11], [12], [13], [14] have identified clusters highly relevant for prostate cancer. Here we have expanded on this aspect by documenting a significant downregulation of a cluster of miRs, exclusively in PCa cells associated with adverse clinical outcome (G1 cells). This cluster comprises miR-34a, the first miR identified as a regulator of the SIRT1 deacetylase [15] a critical epigenetic controller of aging and tumorigenicity [16].

Of note eNOS and NO have also been involved in the aging process, a relevant observation since aging is considered an independent risk factor in several pathological conditions. During aging, eNOS is often deregulated and the usual NO biosynthesis transformed to production of free radicals. This effect contributes to DNA damage and genomic instability providing a favorable ground for cancer development. Indeed eNOS has been recently associated to maintenance of pancreatic cancer [4] and to progression of PCa [1], one of the most common cancer in the elderly. Interestingly, the role of eNOS during the aging process is strictly linked to the function of SIRT1. In physiological conditions, SIRT1 activates eNOS by deacetylation. Aging, by impairing SIRT1 function determines a reduced glucose metabolic efficiency as well as a reduced production of appropriate NO levels, thus deteriorating the intracellular environment.

These premises together with our original finding that the NO pathway represents a “*primum movens*” of a transcriptional program promoting the acquisition of an aggressive phenotype in PCa cells, and that the nuclear translocation of eNOS significantly affects chromatin remodeling of a specific subset of PCa prognostic genes [17], has prompted us to investigate the full potential of this key signaling molecule as master gene in the progression of prostate cancer. Our aim and challenge were to unmask the function of eNOS on a genome-wide scale by a ChIP-Sequencing approach, with the hope of unveiling a general mechanism associated with the presence of nuclear eNOS in prostate tumors. Here we present experimental evidence that defines a molecular circuitry that contributes to aggressive and metastatic PCa and can be modulated by eNOS or SIRT1 inhibitors with potential impact on current therapies for PCa.

## Methods

### Hormones and Inhibitors

17β-Estradiol (E2 Sigma), NG-nitro-L-arginine methyl ester (L-NAME; Alexis), TSA (Sigma-Aldrich), MS275, MC1568, sirtinol, resveratrol were a kind gift of Antonello Mai (Rome, Italy).

### Antibodies

anti–ERα (HC-20 [Santa Cruz Biotechnology]; anti–ERβ (L-20 [Santa Cruz Biotechnology], anti-eNOS (eNOS/NOS Type III [BD Biosciences]; abcam, Cambridge, UK and Cell Signaling, MA,USA), anti-HDAC1 (abcam, Cambridge, UK and Sigma-Aldrich, MO, USA), anti-HDAC2 (Santa Cruz Biotechnology, CA, USA), anti-HDAC3 (Santa Cruz Biotechnology, CA, USA), anti-HDAC4 (abcam, Cambridge, UK and Santa Cruz, CA, USA); anti-HDAC5 (abcam, Cambridge, UK) anti-SIRT1 (abcam, Cambridge, UK); anti-IgG (Santa Cruz Biotechnology, CA, USA), anti-5-methylcytidine (Eurogentec, Seraing, Belgium), anti-α-actin (Sigma-Aldrich), and anti-HSP70 (StressGen Biotechnologies, San Diego, CA).

### Cell Culture

HUVEC and LNCaP cells were cultured as described [1], [5]. Primary prostate cancer cultures were obtained from freshly explanted prostate cancer specimens upon approval of the Institutional Ethical Committee as described [17]. Immortalized PCa-cells were obtained by transduction of hTERT and SV40 large T antigen as described [1]. Clinical data of patients included in the present study have already been reported elsewhere [17]. The outcome of patients (survival, metastasis, local and/or biochemical recurrence) was followed up to December 2012 (observation period, July 2002 to December 2012). Bad prognosis group (G1) of patients with PCa was defined by the presence of biochemical/local recurrence, metastasis, or disease-specific mortality, and Good prognosis group (G2) was defined by complete remission with surgery alone. Cell lines derived from patients have been assigned correspondingly to the G1 (C1IM, C11IM, C13IM, C19IM, C27IM, C43IM, C45IM) or G2 (C14IM, C24IM, C25IM, C35IM, C38IM, C39IM, C40IM, C41IM) phenotypes.

### Transfections, Cell Extracts and Western Blot

Transient transfections were performed by the Jet Pei ™ technique (Poly Plus Transfection). eNOS vector encoding S1177A was a gift from C.M. Counter (Duke University Medical Center, Durham, USA) [4]. Total extracts for Co-IP immunoprecipitation were obtained with USA Buffer (Tris-Hcl 50 mM pH = 7.5; EDTA 5 mM; NaCl 250 mM; Triton 0.1%; NaF 50 mM ), nuclear and cytoplasmic fractions were obtained as previously described [18].

### Treatments

Cells were treated with 10^−7^ M E2, 5 mM L-NAME, 500 nM TSA, 500 nM MS275, 10 µM MC1568, 10 µM sirtinol, 25 µM resveratrol, alone or in combination for the times indicated in figure legends. At least 72 hours prior to experimental use, the cells were switched to medium supplemented with hormone-deprived serum [19].

### ChIPs and Re-ChIPs

ChIP and re-ChIP assays from cultured cells were performed as described [1] using specific antibodies to ERβ, eNOS, SIRT1, HDAC1, HDAC 3, HDAC 4. Negative controls were absence of antibody (NoAb) or normal IgG. Analysis of methylation using antibody anti-5-methylcytidine was performed as described in [5]. DNA fragments were recovered and analyzed by quantitative Real-time PCR as described [1]. Primers are presented in the supplemental section.

For ChIP-sequencing, ChIPs were performed as described [1], [6] with modifications. Briefly, chromatin solutions were prepared by sonication using Bioruptor UCD-200 (Diagenode) to obtain DNA fragments between 150–500 bps in length. Pre-clearing of chromatin solution was performed with protein G-agarose (Pierce) and recovery of immune complexes with protein G saturated with BSA at a final concentration of 1 µg/µl. Immunoprecipitated and input DNA samples were dissolved in double distilled H_2_O. Validation of DNA prior to sequencing was performed by qPCR using primers specific for the hTERT and pS2 promoters (see Supplemental section).

### Library Preparation, ChIP-Sequencing and Bioinformatic Analysis

NGS library preparation and SOLiD sequencing were performed at Genomnia. DNA samples in 50 µl of Tris-HCl 10 mM, EDTA 1 mM, pH 8 were sheared using the Covaris™ S2 System, with the following settings: duty cycle 10%, intensity 5%, cycles/burst 200, cycle time 60 s, number of cycles 3. DNA size after shearing, checked on Bioanalyzer, was 25–400 nt with a peak around 140 nt. Sheared DNA (50 ng) was end–repaired and ligated using Quick ligation kit (New England Biolabs) to 27 pmoles of multiplex P1 adaptor and 27 pmoles of one (different for each ligation) of the bar-coded multiplex P2 double-stranded adaptors (SOLiD™ Fragment Library Oligos Kit, Applied Biosystems pn 4401151). Samples were incubated at room temperature for 10 minutes, purified using the Agencourt® AMPure® Kit, and nick-translation was performed on non-ligated 3′-ends. Finally library molecules were amplified by PCR for 15–17 cycles and purified using the Agencourt® AMPure® Kit as described before. Samples were quantified using Qubit dsDNA HS or BR kits and checked on the Bioanalyzer (Agilent Technology) using a DNA 1000 chip. To obtain the binding and the clonal amplification of library fragments on the surface of sequencing beads, the 8 pooled DNA libraries were added to the emulsion PCR reaction performed according to the manufacturer’s instructions (Applied Biosystems). After amplification, the emulsion was broken with butanol, beads were enriched for template positive beads, 3′-end extended and covalently attached onto one sequencing slide, and sequenced using standard settings on the SOLiD system version 3.5 to produce 50 nucleotide long reads.

### Mapping of Sequencing Data

Mapping of the Color Space sequencing reads to the reference genome (UCSC Homo sapiens hg19 from UCSC) was performed with the Lifetech Lifescope 2.5.1 bioinformatics software suite, after “a priori” error correction with the SAET procedure. The resulting alignment files (pairwise Input and Experiment) in standard.bam format were analyzed for peak calling directly with the MACS software version 1.4.1 [20]. In addition, the.bam alignments were converted to the.bed format with the bamToBed bedtools utility and used for peak calling with the SICER analysis software [21] in order to take into account the heterogeneous nature of these DNA - protein interactions which may include quite long areas of interaction. All intersections between MACS and SICER bed files and genome-wide features was performed with the bedTools v2.17.0 software suite (http://code.google.com/p/bedtools/).

### Peak Identification

Exploratory data analysis of the sequencing and mapping results, of the MACS peak quality, of the relative annotation and further peak calling were performed with the commercial Integromics SeqSolve bioinformatic software suite. Correlation of MACS and SICER peaks (Experiment *versus* Input) with known NCBI RefSeq gene structure and annotation was performed with in-house Genomnia perl scripts or with the ChIPpeakAnno Bioconductor library, version 2.10 [22]. Differential peak analysis was performed with the SICER-df.sh routine of the SICER software or with the Bioconductor DiffBind library [23], complemented by in-house Genomnia perl scripts. Removal of open chromatin (false positives) regions was performed using “A comprehensive collection of signal artifact blacklist regions in the human genome”, Anshul Kundaje, ftp://encodeftp.cse.ucsc.edu/users/akundaje/rawdata/blacklists/hg19/). Statistical test associated with sequence features (evaluation of TSS and peak length mean differences) were evaluated with the Welch Two Sample t-test in the R version 2.15.1 statistical language. Sequence-linked statistical analyses, including kernel tag density calculations, were performed with the appropriate statistical routines and libraries of the R version 2.15.1 statistical language (http://www.r-project.org/). Short-read sequencing data and the associated experiment information have been deposited at the EBI ArrayExpress database (http://www.ebi.ac.uk/arrayexpress/) with the Accession Number E-MTAB-1204.

### SIRT Activity

Enzymatic activity was evaluated with HDAC assay kit (Upstate) according to manufacturer’s instruction using 40micrograms of total extracts.

### Confocal Microscopy

Confocal analysis was performed as previously described [1]. Sample were analysed with a Zeiss LSM510 Meta Confocal Microscope with 63x magnification. For each samples 10 independent fields were analyzed and representative images are shown. Colocalization mask was obtained by LSM510 software to produce images containing exclusively colocalized regions.

### Exiqon Microarrays and Data Analysis

Total RNA purification, including miRNAs, was performed using the miRNeasy kit (QIAGEN) and samples were stored immediately at −80°C. RNA quantification and integrity was assessed using Nanodrop and Agilent 2100 Bioanalyzer. Only samples with a RNA integrity number (RIN) >8,0 were taken for analysis. A total of 500 ng RNA from sample and reference was labelled with Hy3™ and Hy5™ fluorescent label, respectively, using the miRCURY™ LNA Array power labelling kit (Exiqon, Denmark) following the procedure described by the manufacturer. The Hy3™-labeled samples and a Hy5™-labeled reference RNA sample were mixed pair-wise and hybridized to the miRCURY™ LNA Array version 5^th^ Generation (Exiqon, Denmark), which contains capture probes targeting all miRNAs for human, mouse or rat registered in the miRBASE version 16.0 at the Sanger Institute. The hybridization was performed according to the miRCURY™ LNA array manual using a Tecan HS4800 hybridization station (Tecan, Austria). After hybridization the microarray slides were scanned and stored in an ozone free environment (ozone level below 2.0 ppb) in order to prevent potential bleaching of the fluorescent dyes. The miRCURY™ LNA array microarray slides were scanned using the Agilent G2565BA Microarray Scanner System (Agilent Technologies, Inc., USA) and the image analysis was carried out using the ImaGene 9.0 software (BioDiscovery, Inc., USA). The quantified signals were background corrected **(**Normexp with offset value 10 [24] and normalized using the global Lowess (LOcally WEighted Scatterplot Smoothing) regression algorithm. Differential expression of miRNAs between groups was performed using a t-test one-tail after which a *p* value <0.05 was considered statistically significant. A total of 52 miRs were used to generate a heatmap where red and green colors indicate high and low expression respectively. A two-way supervised clustering analysis was performed using Pearson’s correlations and Ward’s criteria as a linkage rule. C27IM cell line was hybridized twice with correlation = 0,92. Microarrays data have been deposited in the Curie database at http://microarrays.curie.fr/, login username and password are available upon request.

### Mature miRNA and pri-miR Detection

Reverse transcription was performed according to the manufacturer’s protocol using TaqMan method (Applied Biosystems, Foster City, CA, USA). Real-time PCR was performed three times in duplicate on an ABI Prism 7500 or 7900 HT Sequence Detection System (Applied Biosystems). Relative amount of each mature miR or pri-miR was measured as fold change using the 2^−ΔΔCt^ method (RNU6B or RNU19 and β−actin or GAPDH served as endogenous control, respectively).

### Statistics

Statistical analysis was performed using Prism 2.01 statistical software (GraphPad). Differences among subject groups were assessed by 2-tailed Mann-Whitney U test and 1-tailed Student’s t-test. A 95% confidence interval (P<0.05) was considered significant. Data are represented as box plots charts (boxes show medians and upper and lower quartiles of the data and whiskers indicate minimum and maximum values), as mean ± SEM or as fold of induction (+/− treatment), as indicated in figure legends.

## Results

### Genome-wide Profile of eNOS-binding Events in Prostate Cancer Cells

We previously documented that in prostate cancer cells eNOS translocates to the nucleus in response to estrogen, a process inhibited by anti-estrogens [5]. Here we show by confocal microscopy that this estrogen-dependent re-localization is also efficiently prevented by L-NAME, an inhibitor of eNOS (**Figure S1**). This finding strongly supports a causal relationship between eNOS activity, NO production and estrogen signaling.

Our open questions were *i.* how does eNOS play a role in prostate cancer under basal conditions or in response to estrogens and, by extension, in the estrogen-dependent transcriptional program associated with prostate cancer aggressive phenotype?, and *ii.* Does the nuclear eNOS function involve molecular interactions with proteins able to modify the chromatin structure and alter the transcriptome in estrogen-responsive prostate cancer cells? To address these questions chromatin immunoprecipitations coupled to massive parallel sequencing (ChIP-seq) were performed before and after treatment with 17β-estradiol (E2, 10^−7^M) in two cell lines: *i*. C27IM cells derived from a primary prostate cancer with an aggressive phenotype and well characterized by immunophenotype, cytogenetic markers, growth and colony formation, gene amplification, mRNA gene and miR profile [1], [17], and *ii*. in LNCaP cells, a human prostate cell line derived from a lymph node metastasis [25], thus representative of the “metastatic” phenotype. The retained responsiveness of these cells to sex steroid hormones, both androgens and estrogens [19], [26], renders them an optimal control for a hormone-responsive primary tumor aggressive but not yet metastatic.

The goal of our study was to identify, in the prostate microenvironment, the primary transcriptional targets of E2 signaling associated with eNOS. We focused on a short hormonal treatment (45 min) on the basis of previous studies by us and others that clearly indicated this timing as optimal for following the primary and immediate effects of E2-dependent transcription, prior to the activation of secondary targets [1], [6], [19], [27], [28].

eNOS ChIP-seq was conducted and eNOS-associated DNA regions (peaks) were identified using two algorithms, MACS 1.4.1 (Linux version or as incorporated in the commercial software SeqSolve ™ from Integromics) and SICER v1.1, to minimize peak caller bias and to consider the ‘extended’ nature of the interaction of eNOS/ER complexes with chromatin [20], [21]. Differential analysis of called peaks or extended regions (islands) was also performed with two methods, comparing and intersecting the results, for the same reasons (see Methods and [22], [23], [29], [30]).

The number of sequencing reads and eNOS-binding events for each cell line, untreated or exposed to E2_,_ are shown in **Table S1**. Under both conditions, the eNOS-containing DNA peaks were found widely distributed across the genome with conserved hyperdensity regions, as shown superimposed to the human chromosome ideograms for both cell lines (**Figure S2**). This pattern, reminiscent of the genomic distribution of ERE sites described by Carroll *et al.* [31], supports our previous findings of the existence of eNOS/ER complexes [1], [5], [6].

**Figure 1:**
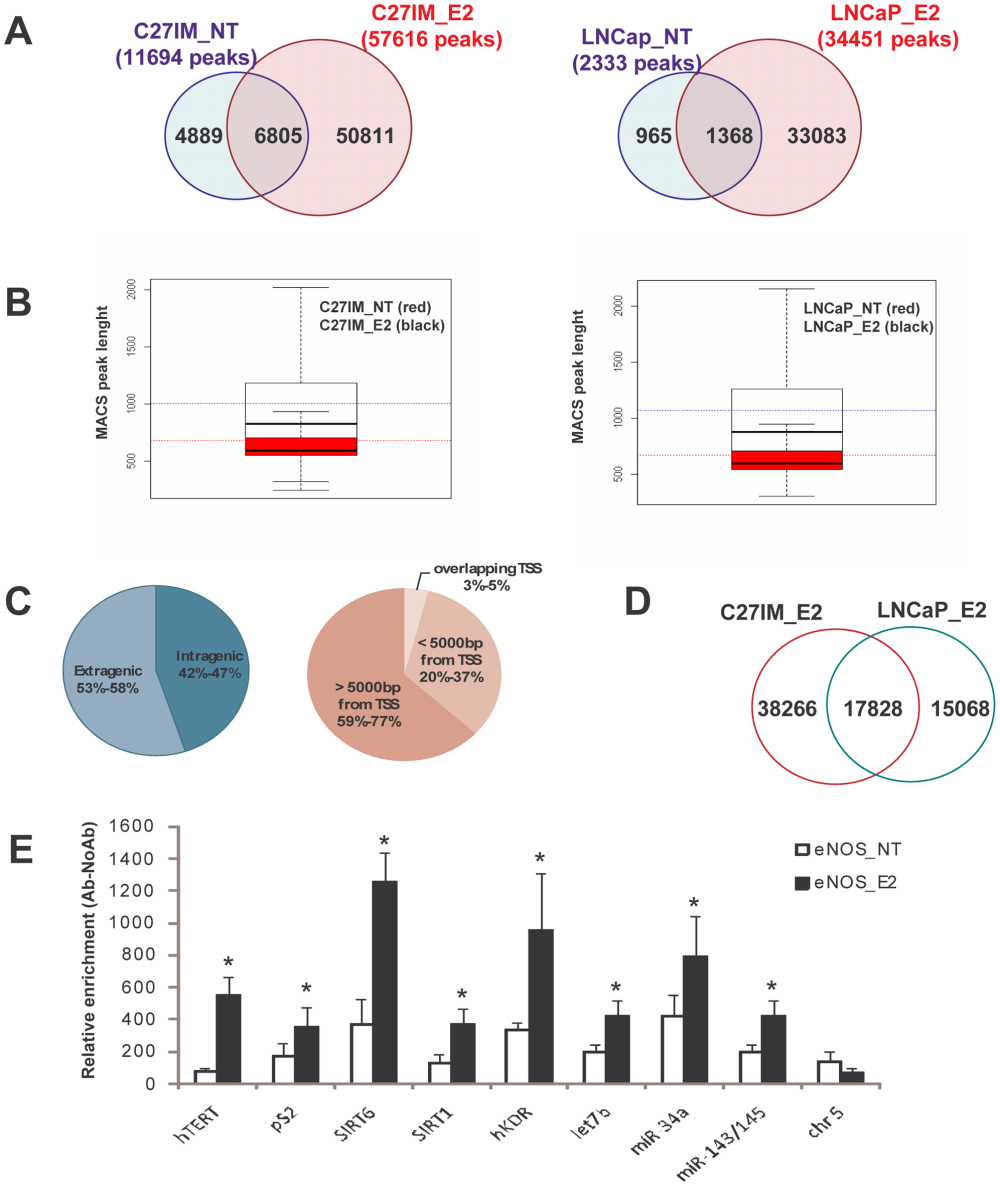
Global overview of eNOS-recruitment by ChIP sequencing and genome-wide changes mediated by estradiol. A) Venn diagram of regions displaying eNOS-recruitment in the absence (NT) or presence of estradiol (E2). Number of discrete genomic eNOS-recruitment peaks identified by ChIP-Seq in C27IM_NT and C27IM_E2 (left), LNCaP_NT and LNCaP_E2 (right) using MACS analysis (FDR<0,1 and P value p<1e-5). Overlap between peaks in NT and E2condition was determined using threshold of 1 nt. B) Length distribution of eNOS peaks in C27 IM_NT, C27 IM_ E2 (left) and LNCaP NT, LNCaP E2 (right). Data are presented as superimposed box plots of peak lenghts for E2 treated (black) or untreated (gray). Black line is the E2 peaks mean length, gray line is the NT peaks mean length. p<2.2e-16 NT vs E2. C) Pie chart of eNOS-peaks distribution in intra/extragenic regions (left) or of distance from TSS (right). Numbers in percentage represent min-max values in each category. D) Venn diagram of MACS-peaks in C27IM_E2 and LNCaP_E2. Overlap between eNOS peaks was determined using threshold of 1 nt. E) Validation by quantitative PCR of ChIP-Seq eNOS-peaks. eNOS binding was monitored in 9 genomic regions, specificity was assessed in an “empty” region (region without eNOS peaks) of chromosome 5. Data represent mean+/−SEM of 3 independent experiments. *p<0,05 eNOS_E2 vs eNOS_NT.

We identified by MACS peak call analysis in C27IM and LNCaP cells, respectively 12,034 and 2,344 eNOS-associated peaks before E2 treatment, and 57,802 and 34,560 thereafter (**Table S2**). Of note, the removal of open chromatin regions known to generate false positives in ChIP-seq experiments (the so-called “ultra-high signal artifact regions”) left substantially unchanged the results in terms of peak number: 11,694 and 2,333 in untreated C27IM and LNCaP cells and 57,616 or 34,451 in C27IM and LNCaP cells upon E2 treatment (**Table S2 and** **Figure 1A** **)** (see Methods and [32].

Clearly upon estrogen treatment the number of peaks increased significantly (4.8- and 14-fold in C27IM and LNCaP cells, respectively) indicating a specific hormone-dependent eNOS re-localization along the genome. A quantitative sequence comparison of eNOS-associated peaks before and after E2 treatment revealed the existence of overlapping peaks between the two conditions, (6,805 common peaks in C27IM and 1,368 in LNCaP cells (**Figure 1A**). A corresponding overlap of 5,190 and 1,630 genes in C27IM and LNCaP cells, respectively, was also found for eNOS-peaks associated with the nearest gene as annotated in the NCBI RefSeq database incorporated in the UCSC Genome Browser (http://www.ncbi.nlm.nih.gov/RefSeq/), (data not shown). The overlapping peaks (and associated genes) thus represent a sub-set of eNOS-bound regions that are not responsive to estradiol treatment, suggesting eNOS interactions with proteins others than ER.

On the other end, the majority of peaks are sensitive to E2, resulting in induction of *de-novo* eNOS genome binding (50,811 peaks in C27IM and 33,083 in LNCaP) or detachment (4,889 peaks in C27IM and 965 in LNCaP). Of interest, in both cases, multiple eNOS MACS peaks induced by estradiol exist *per* gene. In E2-treated cells most eNOS target genes were bound once or twice, about 5% were bound 3 times, and about 9% were bound 4 or more times (**Table S3)**. Moreover, E2 stimulation altered the distribution of eNOS as indicated by significantly increased peak length after E2 treatment suggesting a DNA-eNOS/ER complex stabilization following hormonal treatment (**Table S2 and** **Figure 1B** **)**.

Distribution of eNOS-peaks relative to the nearest TSS obtained using Kernel tag density analysis revealed that *i.* their frequency is centered on the TSS and decreases on both sides with a clear asymmetry toward the intragenic areas (**Figure S3A**) and *ii*. E2 treatment broadened the global area covered by the peaks in C27IM and, to a less extent, in LNCaP cells, as shown by using a 10.000 bp window, although the peaks showed a significant enrichment around TSS following E2 stimulus (**Fig. S3B**).

Remarkably, the global peak distribution in annotated genomic regions was similar in both experimental conditions (+/− E2), with a slight prevalence (53–58%) of peaks localized in extragenic regions, and the remainder in intragenic regions, in particular within introns (**Figure 1C**). As shown in Figure 1D, there was considerable overlap between peaks induced by E2 in C27IM and LNCaP cells.

Correlation between number of sites identified by MACS and SICER in both C27IM and LNCaP cells revealed a substantial concordance (see Methods), further validated by the results of ChIP assay shown in **Figure S4**. For example hTERT, an estrogen target gene, with several known EREs [1], [19], [33], [34] displays MACS peaks and E2-increased SICER islands in correspondence to sites well characterized by ChIP-qPCR (**Figure S4A**); pS2 (TFF1), a classical estrogen target gene, exhibits E2-increased SICER-derived islands at sites amplified by ChIP-qPCR (**Figure S4B**); GSTP1, a gene silenced by the ERβ-eNOS complex in the absence of ligand [5] shows MACS peaks in correspondence to sites amplified by ChIP-qPCR, exclusively in the unstimulated condition (**Figure S4C**).

Moreover, the matrix of pairwise correlation depicting DNA occupancy on the basis of the MACS peak caller score and coordinates (**Figure S5A**) as well as the hierarchical cluster analysis of binding affinity (heatmap) showing affinities for differentially bound sites calculated from read count data and MACS peak coordinates (**Figure S5B**) revealed that binding sites clustered first in response to E2, and then according the cell type. A total of 692 E2 vs NT differentially represented peaks (FDR <0.05) were identified, of these 287 with a positive fold change and the remainder with a negative one.

**Figure 2:**
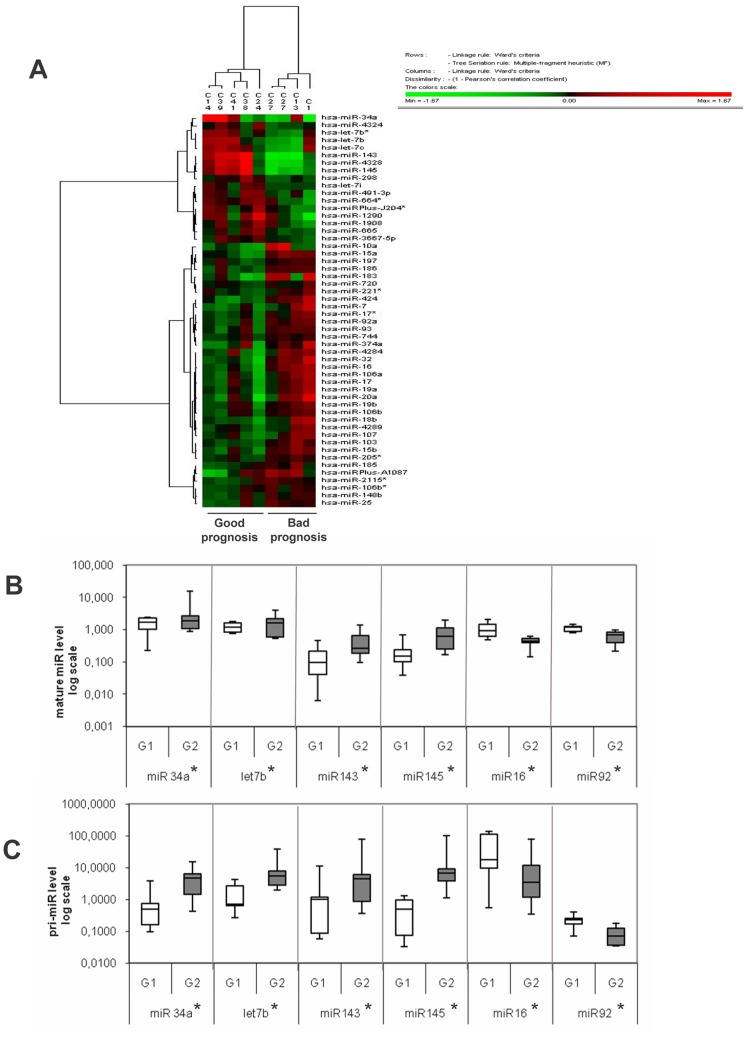
Prognostic miRNA signature. A) Supervised cluster analysis of microRNAs profiling (Exiqon Array) for the two groups of patients defined by recurrence status (Good or Bad prognosis). B) Validation by quantitative real time PCR of differential miRNAs levels in the G1 (Bad prognosis n = 7) and G2 (Good prognosis n = 8) groups, *p<0,05 G1 vs G2. C) Differential level of primary transcripts (pri-miR) in G1 (n = 7) and G2 (n = 8), *p<0,05 G1 vs G2. Data are represented as box plot on a logarithm scale.

Lastly, we validated the eNOS ChIP-Seq data enrichment observed upon estradiol treatment. The E2 effect was monitored in C27IM cells, untreated (NT) or E2-treated, using anti-eNOS antibody and ChIP-qPCR on eight gene promoters previously identified or derived from microRNA profile analysis (**Figure 1E** **and** **Figure 2A** below) [1]. Our results reveal a significant correlation between the presence of eNOS-peaks, as emerging from ChIP-Seq data set upon estrogen treatment (**Figure S4** and *data not shown*), and the estrogen-induced recruitment of eNOS onto the same genomic regions as assessed by traditional ChIP-qPCR. Enrichments were normalized to the absence of antibody (noAb), or an unrelated antibody (Ab IgG). Specificity of the ChIp-Seq was ensured using primers amplifying a genomic region within chromosome 5 lacking eNOS peaks and simultaneously showing the absence of eNOS recruitment by classical ChIP-qPCR.

#### Cluster analysis of miRNAs pattern in PCa cells

With the aim of differentiating lethal and non-lethal prostate cancer and ultimately improving the clinical outcome of subjects diagnosed with the disease, and based on the observed eNOS association with regulatory regions of annotated pri-miR (**Table 1**), we combined the ChIP-Seq approach with miR profiling in a subset of our PCa-derived cell populations [17] using an Exiqon platform.

**Table 1:** Number of eNOS peaks encompassing 50 kb genomic regions upstream of annotated pre-miRs.

Down-Regulated miRs^a^	C27IM NT	C27IM E2	LNCAP NT	LNCAP E2
hsa-miR-34a	1	3	–	3
hsa-miR-4324	2	2	–	1
hsa-let-7b	2	3	–	1
hsa-let-7c	–	1	–	–
hsa-miR-143	–	1	–	–
hsa-miR-4328	3	–	–	–
hsa-miR-145	–	2	–	–
hsa-miR-298	3	5	–	2
hsa-let-7i	–	–	–	2
hsa-miR-491-3p	–	–	–	–
hsa-miR-664*	–	–	–	–
hsa-miR-1290	–	10	–	–
hsa-miR-1908	–	4	–	3
hsa-miR-665	–	2	–	–
hsa-miR-3667-5p	–	–	–	–
*TOTAL*	*11*	*33*	*0*	*12*
**Up-Regulated miRs** a	**C27IM NT**	**C27IM E2**	**LNCAP NT**	**LNCAP E2**
hsa-miR-10a	2	10	–	7
hsa-miR-15ahas-miR-185	–	–	–	–
hsa-miR-197	–	2	–	3
hsa-miR-186	–	1	–	–
hsa-miR-183	–	8	–	5
hsa-miR-720	–	–	1	–
hsa-miR-221*	–	–	–	–
hsa-miR-424	11	1	–	–
hsa-miR-7	1	5	1	2
hsa-miR-92a	–	–	–	–
hsa-miR-93	–	–	–	–
hsa-miR-744	–	–	–	–
hsa-miR-374a	7	1	–	–
hsa-miR-4284	–	6	–	2
hsa-miR-32	–	–	–	–
hsa-miR-16	1	2	–	–
hsa-miR-106a	6	1	–	–
hsa-miR-17	–	–	–	–
hsa-miR-19a	–	–	–	–
hsa-miR-20a	–	–	–	–
hsa-miR-106b	1	2	–	1
hsa-miR-18b	–	–	–	–
hsa-miR-4289	–	–	–	–
hsa-miR-107	–	–	–	–
hsa-miR-103	4	–	2	–
hsa-miR-15b	–	–	–	1
hsa-miR-205*	–	–	–	–
hsa-miR-185	2	4	–	2
hsa-miR-2115*	–	–	–	–
hsa-miR-148b	–	–	–	1
hsa-miR-25	1	–	–	1
*TOTAL*	*36*	*43*	*4*	*25*

a as shown in G1-PCa cells associated with bad prognosis (see miRs levels in cluster of Figure 2).

The miR profile (Fig. 2A) confirmed the differential miR expression originally described in prostate cancer histological samples [12], thus validating again our ex-vivo cellular model [1], [5], [17]. In particular, a clear down-regulation of a specific cluster of miRs (e.g. miR-34a, let-7c, miR-143/miR-145) was observed in PCa-cells derived from the bad prognosis group, in agreement with recent reports on the role of these miRs in PCa progression [11], [35], [36], [37]. These data substantiate the notion that reduced expression of these specific miRs may be involved in the acquisition of a more aggressive phenotype.

Since in previous work we had identified a prognostic transcriptional signature characterized by a wide dysregulation of several messenger RNAs specifically associated with PCa-cells with aggressive phenotype [17], miR-array hybridizations were performed in 8 (out of the 22 originally analyzed) PCa-derived cell lines, 5 from patients with non-recurrent (Good prognosis, G2) and 3 with recurrent prostate cancer (Bad prognosis, G1), including the C27IM cell line subjected to ChIP-Seq analysis. The comparison between the two groups identified 52 miRs with significant changes in expression that were used to draw a clustering analysis that: 1) readily separated the two groups of patients (Good *versus* Bad) and 2) clearly showed divergent expression (down-regulated *versus* up-regulated) associated with opposite clinical outcome (**Figure 2A**). Of interest, a number of these miRs have previously been reported to show similar down- or up-regulation in prostate cancer compared to normal prostate tissue, and some of them (e.g., miR-34a, miR-16, miR-145, miR-205) have been specifically linked to prostate cancer metastasis [35], [38], again supporting the validity and reproducibility over time of our *ex vivo* experimental model. Validation of data derived from the microarray platform has been performed using *q*RT-PCR by evaluating expression of 6 different miRs, either up or down-regulated, at the mature and primary transcript level (**Figure 2BC**). We presumed that differential miRs expression between G1 and G2 cells could be due to a different transcriptional regulation, an hypothesis confirmed by the analysis of the primary transcripts.

#### Identification and validation of eNOS peaks in the regulatory regions of miRs associated with opposite outcome

To determine whether there was a correlation of the miR profile with data deriving from the ChIP-Seq experiments, we systematically searched for eNOS-bound peaks along the genomic regions upstream of the annotated pre-miRs (according to UCSC annotated Hs genome hg19), focusing, in particular, on those downregulated in the bad prognosis group. Of note, a consistent number of eNOS peaks, among the downregulated and upregulated miR clusters, were found 50 kb upstream of pre-miRs, including major putative transcriptional regulatory regions (**Table 1**). Calculating a window of 50 Kb around any given location in the human genome and a totally random distribution of the peak population (depleted from the hyper chippable regions), we would expect 0.195 peaks for C27IM NT; 0.95 peaks for C27IM E2; 0.039 peaks for LNCAP NT and 0.575 peaks for LNCAP E2. Thus, the frequency of peaks upstream of annotated pri-miRs shown in **Table 1** denotes a clear enrichment.

Of interest, although the overall number of peaks was higher in C27IM as compared to LNCaP cells, addition of E2 further increased peak numbers suggesting that recruitment onto these miR regulatory regions is essentially driven by the ligand-activated estrogen receptor. However we cannot exclude, as alternative mechanism, the involvement of non-genomic estrogen action [39], [40].

Moreover, this is suggestive of a repressive role mediated by the ligand-activated eNOS/ER complex at least among the “tumor suppressor” miRs. The higher frequency of this event in cells derived from primary tumors is compatible with this being an early molecular step in the progression of prostate cancer.

Among the various miRs differentially expressed in our cell populations we focused on miR-34a because it has been recently demonstrated to be a key negative regulator of prostate cancer stem cells and metastasis [35].

**Figure 3:**
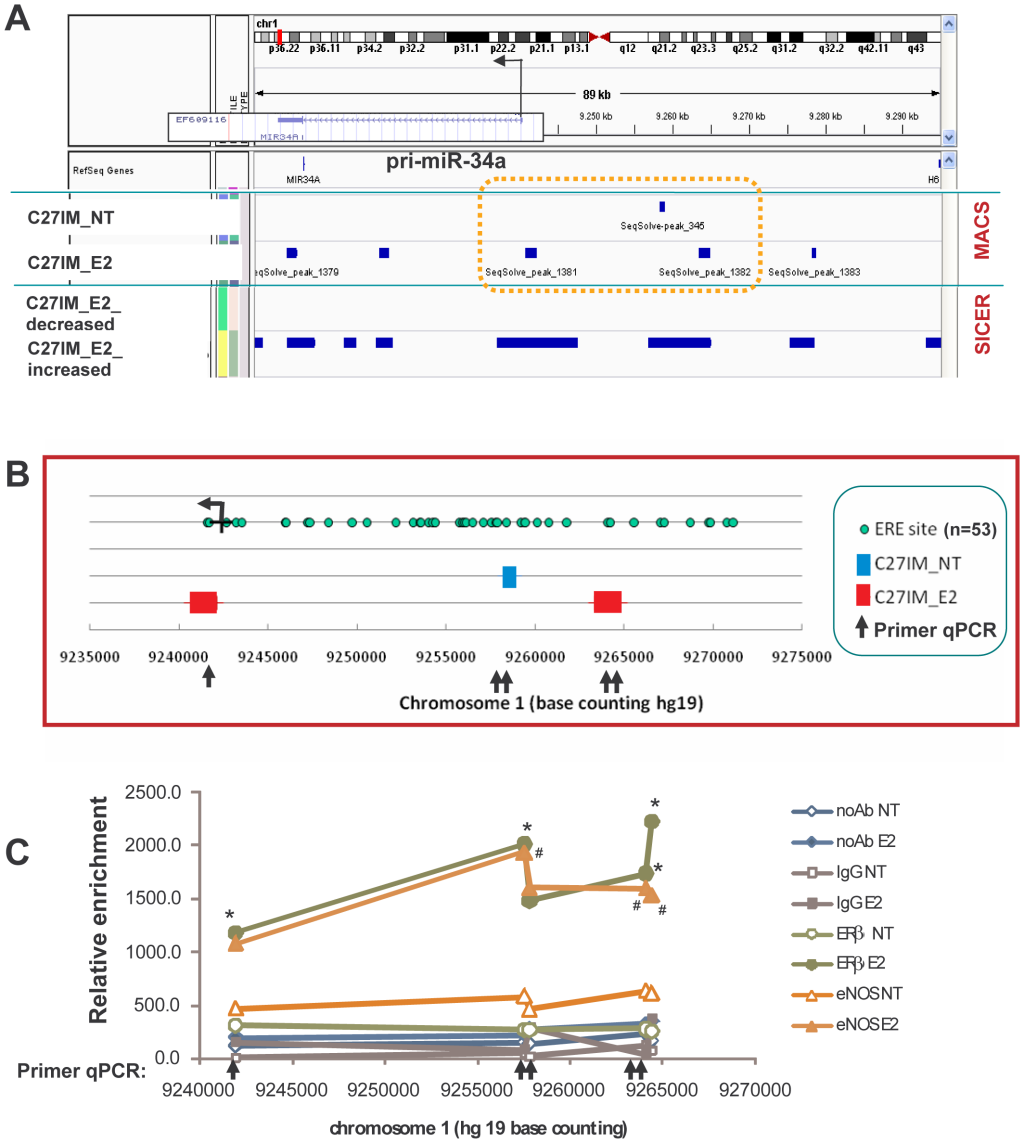
Schematic representation of the miR-34a genomic region and eNOS-peaks identified by ChIP-sequencing. A) Integrated Genome Viewer (IGV 2.1) screenshots showing pri-miR-34a genomic region (TSS is indicated) and eNOS-binding in C27IM, in the presence or absence of estradiol (E2), identified by ChIP-Seq using two different algorithms MACS and SICER. B) Molecular dissection of orange dashed area indicated in A. Locations of EREs identified by MatInspector analysis are shown by green circles, eNOS peaks identified by MACS are shown by boxes (blue C27IM_NT, red C27IM_E2) and primers for ChIP-qPCR by arrows. C) ChIP validation of eNOS peaks and dynamic recruitment of eNOS and ERβ in the regulatory region of pri-miR34a, in the presence or absence of E2. *p<0,05 ERβ E2 vs ERβ NT; ^#^p<0,05 eNOS E2 vs eNOS NT.

**Figure 3A** depicts a cartoon of the pri-miR-34a genomic region and overlapping eNOS-binding identified by ChIP-Seq using two different algorithms, MACS and SICER, in C27IM cells, in the presence or absence of E2. To understand how eNOS collaborates with ER, we first molecularly dissected a specific genomic area (delimited in **Figure 3A** by the dashed area). In addition, a classical peak profile of the indicated eNOS peaks, compared to control input, is provided in **Figure S6**, to certify their validity. Upon screening for ER consensus sequences by using MatInspector, ERE motifs were highly ranked as expected, and 53 EREs (canonical, non-canonical and half site) were found (**Figure 3B** **)**. Based on the location of the peaks, specific primers for ChIP-qPCR were designed to follow the dynamic recruitment of eNOS and ERβ onto the regulatory region of pri-miR-34a, before and after E2. A consistent occupancy by both proteins of the regulatory region of pri-miR-34a was observed in basal condition and was found potentiated upon hormonal treatment, with the highest enrichment of 3.4- and 8.4-fold for eNOS and ERβ over the untreated (**Figure 3C** **)**, thus validating the ChIP-Seq data.

### Effects of Estradiol on the Expression of miR-34a and of its Target SIRT1

**Figure 4:**
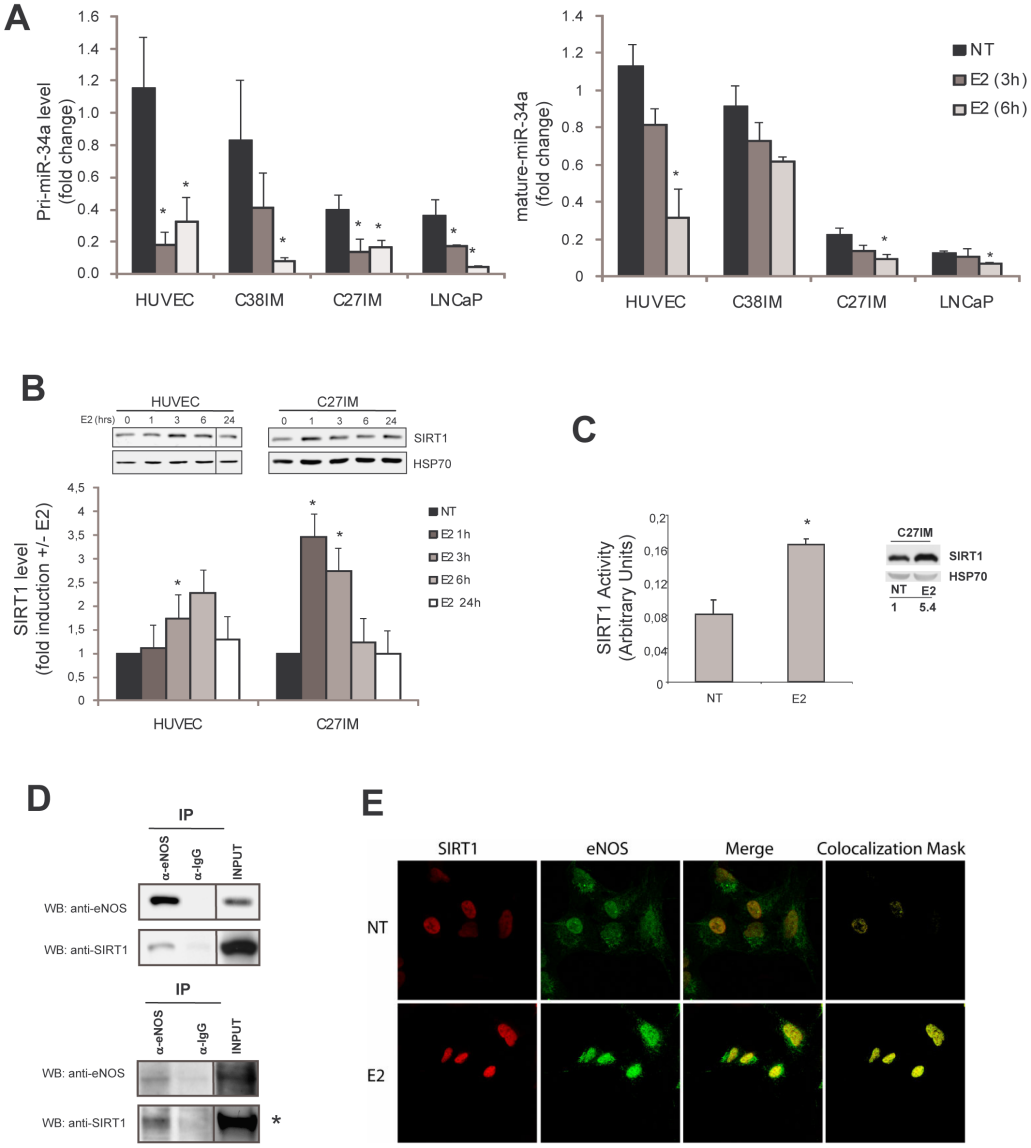
Effect of Estradiol on miR-34a level and its target SIRT1. A) Normal HUVEC cells, PCa-cells from the G2 and G1 groups (C38IM and C27IM, respectively) and LNCaP cells were treated with estradiol (E2) for 3 and 6 hours and levels of pri- (left) and mature (right) miR-34a were analysed by qRT-PCR. Data represent the mean ± SEM of 4 experiments. *p<0,05 vs NT. B) HUVEC and C27IM cells were treated with E2 and SIRT1 level was assessed by western blot. Upper: representative experiments. Black lines indicate samples run in noncontiguous lanes of the same gel. Lower: Densitometric analysis of SIRT1 vs HSP70 level is expressed as fold induction +/−E2. Data represent the mean ± SEM of 3 experiments. *p<0,05 vs NT. C) SIRT enzymatic activity (left) and corresponding level of protein (right) was evaluated in C27IM cultured for 72 h hours in hormone-deprived serum before and treated with E2 for 1 h. *p<0,05 vs NT. D) Co-IP of eNOS and SIRT1 in HUVEC and C27IM cells (upper and lower panel, respectively). IgG served as negative control and cell extract (Input) as positive control. Black lines indicate samples run in noncontiguous lanes of the same gel. *Lower exposure. E) C27IM cells were cultured for 72 h hours in hormone-deprived serum before treatment with E2 for 2 h15’. Cells were stained with antibody to SIRT1 (red) and eNOS (green) and analyzed by confocal microscopy. Nuclear co-localization is evidenced by colocalization mask.

Our findings of repression of a miR subset associated with a PCa aggressive phenotype even in basal condition, and the increase of eNOS peaks upon estrogen treatment, prompted us to evaluate whether E2 itself further affected expression of the constitutively downregulated miR cluster (**Figure 2**). PCa-cells belonging to the good and bad prognosis groups (C38IM and C27IM, respectively), LNCaP and HUVEC cells were treated with E2 for 3 and 6 hours and level of pri-miR-34a (left) and mature miR-34a (right) were analysed by *q*RT-PCR. A significant repression of both forms of miR-34a was observed in all cell lines (**Figure 4A**), more pronounced for the pri-miR than the mature miR, suggesting estrogen regulation at the level of transcription. Of interest, in the unstimulated condition, expression of miR34-a (at both pri-miR and mature level) progressively decreased from normal endothelial to metastatic cancer cells, in agreement with recent reports linking miR34-a silencing with the appearance of prostate cancer stem cells and metastasis [35]. Similar results were obtained with other miRNAs belonging to the same cluster such as let-7b, miR-143, miR-145 (*data not shown*).

Since expression of miR-34a is inversely correlated to that of his target SIRT1, we asked whether E2 treatment could positively affect SIRT1 expression and activity. HUVEC and C27IM cells were treated with E2 versus time. A reproducible induction of SIRT1 protein and activity by E2 in HUVEC andC27IM cells, although with a different kinetics was observed (**Figure 4B and C**).

On the basis of previous reports [41] and of the ability of SIRT1 to deacetylate (hence activate) eNOS, Co-IPs between eNOS and SIRT1 and confocal microscopy analysis were performed. A clear protein-protein interaction was observed in the basal condition in HUVEC and C27IM cells (**Figure 4D**). Moreover nuclear colocalization of both proteins, greatly enhanced upon estrogen stimulation, was observed by confocal analysis in C27IM cells (**Figure 4E**).

### Rescue of miR-34a Estrogen-dependent Repression by SIRT1 and HDACs Inhibitors

**Figure 5:**
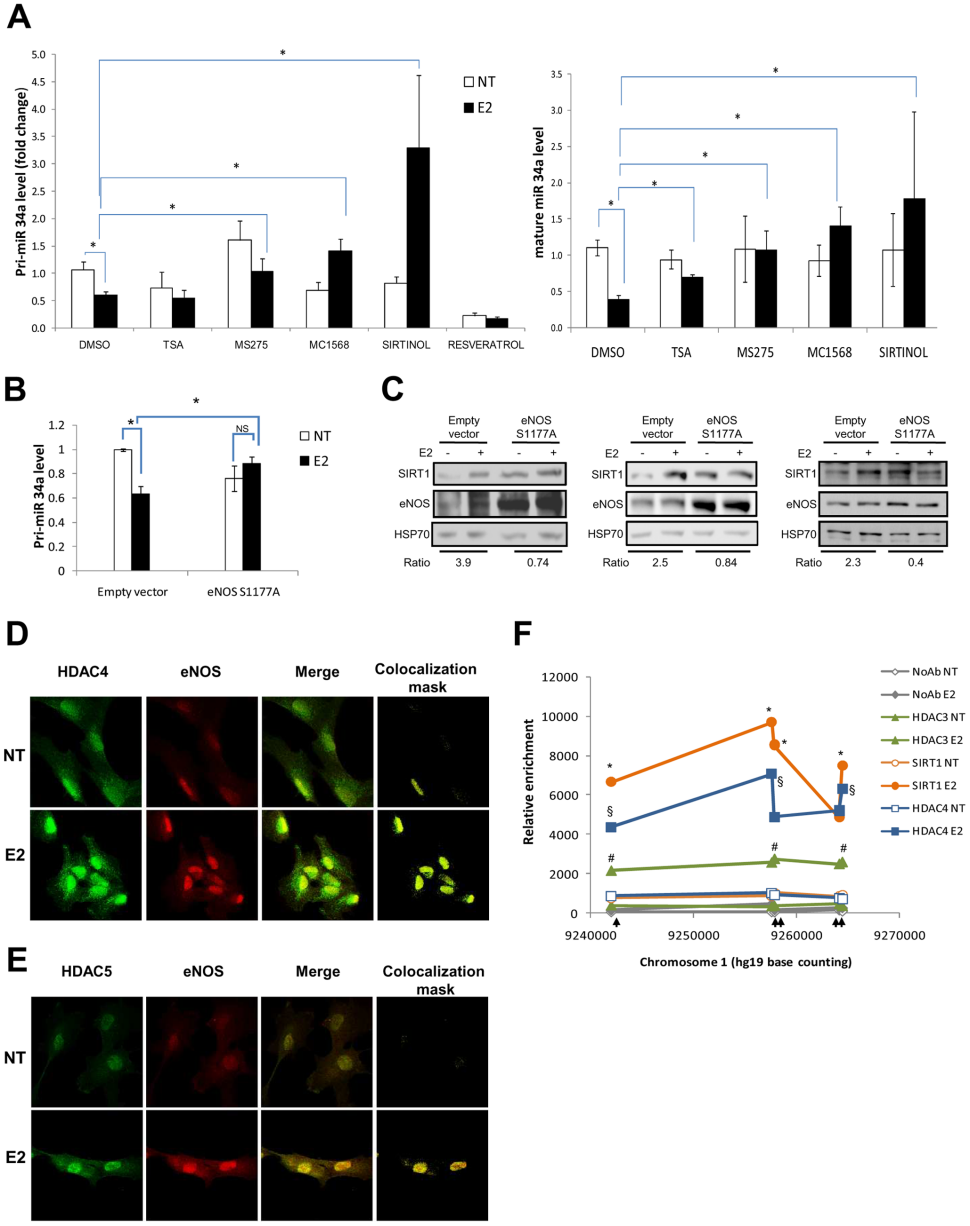
Rescue of estrogen-dependent repression of miR34a by HDACs inhibitors. A) C27IM cells were treated with estradiol (E2) for 3 hours in the presence or absence of inhibitors of deacetylases (TSA, MS275, MC1568), or the SIRT1 inhibitor Sirtinol, or the SIRT1 activator Resveratrol, added 30 minutes before the hormone. Levels of pri-miR-34a (left) and mature-miR34a (right) were analysed by qRT-PCR. Data represent the mean ± SEM of 4 experiments. *p<0,05 B) C27IM were transfected with the eNOS dominant negative mutant (S1177A) or empty vector for 48h in presence or absence of E2 added 1h prior to harvesting. Levels of pri-miR-34a were analysed by qRT-PCR. Data represent the mean ± SEM of 3 experiments. *p<0,05 C) C27IM (left), LNCaP cells (middle) and HUVEC (right) were transfected with the eNOS dominant negative mutant (S1177A) or empty vector for 48 h in presence or absence of E2 added 1 h prior to harvesting. SIRT1 levels were assessed by western blot. Hsp70 served as loading control. Ratio +/−E2 of SIRT1 level normalized with Hsp70 are indicated. D, E) Confocal analysis of eNOS and HDAC4 (D) and HDAC5 (E) in the presence or absence of E2 as described in the legend to Figure 4. F) Dynamic recruitment of class I (HDAC3), class II (HDAC4) deacetylases or SIRT1 analyzed by ChIP in the regulatory region of pri-miR34a in the presence or absence of E2. Arrows represent primers for qPCR as in Figure 3. *p<0,05 SIRT1 E2 vs SIRT1 NT; ^#^p<0,05 HDAC3 E2 vs HDAC3 NT; §HDAC4 E2 vs HDAC 4 NT.

With the aim of deepening our insights into the molecular mechanisms underlying the E2-dependent negative regulation of miR-34a, we treated C27IM cells with several inhibitors: Sirtinol, specific for SIRT1, Trichostatin A (TSA) specific for class I and IIa mammalian histone deacetylases (HDACs), but not class III HDACs (i.e., Sirtuins) [42], or MS275 and MC1568 specific for HDACs I and IIa, respectively. The SIRT1 activator, Resveratrol, was also included as control. **Figure 5A** shows that Sirtinol was effective in preventing the E2-mediated silencing of miR-34a, both at mature and pri-miR levels. MC1568 also rescued miR-34a expression although to a lower extent, whereas MS275 and TSA appeared to be the least efficient As one would expect, Resveratrol was totally ineffective in rescuing the estrogen-dependent miR-34a silencing.

To assess the specificity of our findings and to prove the primary role played by eNOS together with ERβ in mediating this transcriptional regulation, we evaluated miR-34a levels upon overexpression of a dominant negative eNOS mutant, S1177A [4], in C27IM, before and after E2. As expected from the results shown in Fig. 5A, transfection of empty vector did not affect the E2-dependent inhibition of pri-miR34a. On the other hand, the eNOS mutant rescued the E2-dependent pri-miR34a repression (**Figure 5B**) with the consequent relief of the estrogen-dependent SIRT1 induction in C27IM as well as in LNCaP and HUVEC cells (**Figure 5C**). Of note, in the presence of dn-eNOS we observed a stabilization of SIRT1 possibly due to partial reduction of pri-miR34a in the absence of estrogen (**Figure 5B**). In all cases, however, estradiol treatment, which usually increases the intracellular levels of NO, failed to induce SIRT1 expression in this condition. These results reveal the existence of a feedback loop whereby, upon estrogen, SIRT/eNOS regulates transcription of miR-34a which in turn modulates SIRT1 expression.

Overall these findings are highly suggestive of a direct involvement of NAD+-dependent deacetylases such as SIRT1, and to a less extent, of histone class IIa deacetylases such as HDAC4 or HDAC5. To investigate the mechanism by which these molecules participate in the miR-34a estrogen-dependent transcriptional silencing, we performed confocal analysis and ChIP assays in the presence or absence of E2 and analyzed their recruitment onto a long genomic regulatory region upstream of the miR-34a promoter. Confocal microscopy revealed that E2 treatment increased the co-localization of HDAC4/eNOS and HDAC5/eNOS (**Figure 5D, E**). Moreover, since HDAC4 and 5 do not bind directly DNA but acquire this property through their interaction with HDAC3 [43], [44], ChIP assays were performed with antibodies to HDAC 3 and 4 as well as SIRT1 (**Figure 5F**). Occupancy of the miR-34a promoter was observed essentially only after hormonal stimulation, HDAC4 and SIRT1 exhibiting the highest enrichment (with a maximum of 8.4- and 10.6-fold over control, respectively). These data substantiate the presence of hetero-complexes suggesting that the interplay among members of class I, IIa and III contributes to the estrogen-dependent repression of miR-34a in aggressive prostate cancer cells.

## Discussion

In this study we have generated a genome wide profile of eNOS-DNA associations that has revealed an unexpected very wide distribution of the protein. Since eNOS is not *per se* a DNA-binding protein nor a classical transcription factor, this observation suggested to us its association with a variety of chromatin-binding partners. Indeed, we show that eNOS can interact with the genome in a dual-fashion, either by forming complexes with transcription factors that bind specific DNA motifs, or by associating with proteins, such as histone deacetylases, present over large genomic regions. Evidence that eNOS contributes to transcriptional regulation by forming complexes with ERβ and HIFs, detectable on chromatin or in solution, has been provided by our previous work [1].

The existence, under basal conditions or in response to estrogen, of a variety of putative eNOS-targeted genes distributed genome-wide, suggests a key role for eNOS in PCa (**Figure 1A and D**). In agreement with that, gene ontology analysis of over-represented pathways shared by C27IM and LNCaP cells exclusively upon estrogen stimulation (**Table 2**) shows a series of gene pathways involving eNOS in PCa progression, thus revealing an unprecedented scenario for eNOS involvement at the chromatin level in tumorigenesis. Recent data [**45**] have in fact provided evidence supporting eNOS-targeted therapy in clinical oncology.

**Table 2:** Over-represented pathways for overlapping eNOS-peaks in C27IM and LNCaP treated with estradiol.

Name of event	Event identifier^a^	Total number of genes involved in the event	Number of genes in your query mappingto the event	P value^b^
Hemostasis	REACT_604	478	170	7.91E-10
Developmental Biology	REACT_111045	407	166	1.78E-15
Transmembrane transport of small molecules	REACT_15518	432	129	0.001395704
Neuronal System	REACT_13685	290	125	6.60E-14
Axon guidance	REACT_18266	281	119	1.22E-12
Metabolism of lipids and lipoproteins	REACT_22258	315	90	0.02380377
Signalling by NGF	REACT_11061	222	85	5.60E-07
SLC-mediated transmembrane transport	REACT_19118	251	81	0.001023465
Transmission across Chemical Synapses	REACT_13477	189	80	7.29E-09
Platelet activation, signaling and aggregation	REACT_798	205	78	2.20E-06
Neurotransmitter Receptor Binding And Downstream Transmission In The Postsynaptic Cell	REACT_15370	136	54	2.01E-05
Cell-Cell communication	REACT_111155	137	54	2.57E-05
Signaling by PDGF	REACT_16888	122	53	9.92E-07
NGF signalling via TRKA from the plasma membrane	REACT_12056	136	53	4.40E-05
Integration of energy metabolism	REACT_1505	114	49	3.67E-06
Potassium Channels	REACT_75908	99	48	5.51E-08
Signaling by Rho GTPases	REACT_11044	125	47	0.000315471
Rho GTPase cycle	REACT_11051	125	47	0.000315471
Signaling by NOTCH	REACT_299	117	44	0.000482562
L1CAM interactions	REACT_22205	109	42	0.000354716
Signaling by EGFR in Cancer	REACT_115871	110	41	0.000913295
Integrin cell surface interactions	REACT_13552	85	40	1.87E-06
Cell surface interactions at the vascular wall	REACT_12051	92	40	2.05E-05
Downstream signal transduction	REACT_17025	93	40	2.79E-05
Signaling by EGFR	REACT_9417	108	40	0.001209373
Signaling by FGFR	REACT_9470	112	40	0.002647301
Downstream signaling of activated FGFR	REACT_21272	100	39	0.000428773
Signaling by ERBB2	REACT_115755	102	38	0.00139995
Class B/2 (Secretin family receptors)	REACT_18372	88	37	9.91E-05

a as in http://www.reactome.org/ReactomeGWT/entrypoint.html.

b Un-adjusted probability of seeing n or more genes in the event by chance.

These findings are extremely novel with respect to the more established cytoplasmic localization and role attributed to eNOS [46], [47], and reinforce the specific eNOS nuclear function in aggressive prostate cancer proposed by our previous *in vivo* studies on TissueMicroArrays from PCa patients. In fact we have demonstrated that higher expression of eNOS, cytoplasmic and nuclear, was, together with ERβ and HIFs, the most relevant indicator of adverse clinical outcome within a prognostic expression signature [1], [17].

In investigating the effects of estrogen on the transcriptional regulation involving eNOS in PCa we have focused on two genes: miR-34a, whose expression is decreased in PCa, and one of its targets, the class III deacetylase SIRT1 [48], [49]. One mechanism by which miR-34a expression decreases in cancer is through aberrant CpG methylation of its promoter [50]. However, by immunoprecipitation of methylated DNA [5], we observed a low/intermediate level of methylation in 4 PCa-derived cell lines (including C27IM) compared with LNCaP cells (*data not shown*). Thus methylation does not appear to be the only mechanism responsible for miR-34a silencing, at least in PCa cells. We indeed have previously provided evidence of an alternative silencing mechanism attributable to the eNOS/ERβ complex activated by E2 [5].

With regard to SIRT1 it is known that its expression is increased in a variety of human cancers including PCa [48], [51], suggesting a critical role of this protein in tumorigenesis [49]. The crosstalk between SIRT1 and eNOS is determined by the deacetylation of lysines 496 and 506 which reside within the calmodulin binding domain of eNOS that is, in turn, activated by SIRT1 [41]. This effect is generally considered part of the protective and beneficial function of the SIRT1/eNOS axis. As a consequence of its important biological properties SIRT1 has emerged as a drug development target for treating age-dependent diseases. It appears, however, that biologically its main function resides in the optimal balancing between aging and cancer [16].

**Figure 6:**
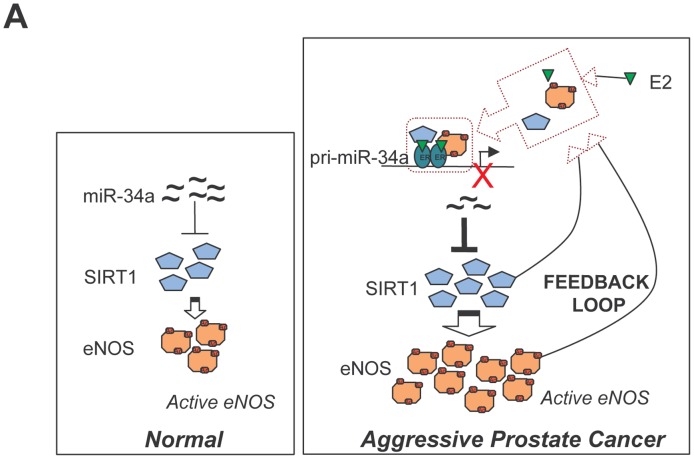
Cartoon of proposed eNOS/SIRT1 interplay in prostate cancer cells in response to estrogen.

Based on our results we propose the following model: in aggressive PCa an abnormal estrogen stimulation, resulting from the decrease of androgen in favor of estrogen during aging [52], results in almost complete silencing of miR-34a. As a consequence, there is a rapid increase in SIRT1 expression that determines, in turn, activation of eNOS. The resulting positive feedback on both proteins (see cartoon in **Figure 6** **)** further represses transcription of miR34-a. Silencing of this miR has already been linked to promotion of prostate cancer stem cells and metastasis [35]. Our contribution reveals the molecular mechanisms responsible for this phenomenon, i.e. its dependence on the transcriptional repressive function of eNOS-containing complexes. Genetic inhibition of eNOS by overexpression of a dominant negative eNOS mutant, interrupts the eNOS/miR-34a/SIRT1 pathway, thus validating our hypothesis (**Figure 5B,C**).

Moreover, the estrogen-dependent repression of miR-34a is prevented by treatment with HDAC inhibitors (**Figure 5A**), the SIRT1 specific inhibitor Sirtinol being the most efficient, revealing a tight link between SIRT1 and eNOS through the SIRT1 regulator, miR-34a [15]. The effects obtained with the HDAC inhibitors favors the view of eNOS as a critical factor participating in chromatin remodeling complexes in association with different classes of histone deacetylases (**Figure 5F**).

Of interest, the small polyphenolic compound, Resveratrol, a classical SIRT1 activator, did not rescue miR-34a expression (**Figure 5A**). This may be explained by an additive effect of Resveratrol on the eNOS/NO pathway given its known role on SIRT1 activation and NO production. On the other end, Resveratrol has structural similarity to the synthetic estrogen diethylstilbestrol and acts as an estrogen receptor partial agonist [53], [54]. These features may provide an alternative interpretation of our data showing that in the presence of this ER ligand, the repression of miR-34a transcription induced by eNOS/ERβ complex cannot be reversed.

In conclusion, we have documented the genome-wide existence of a considerable number of eNOS-DNA associations that define transcriptional active regions, as well as a significant downregulation of a cluster of miRs, among which miR-34a, in PCa cells associated with adverse outcome (G1 cells). These results have revealed a molecular link between eNOS, SIRT1 and miR-34a in the prostate microenvironment. We have also identified, in the same context, novel eNOS-targeted genes (e.g. miRs and their targets) in both untreated and E2-treated cells, a finding that suggests that eNOS may participate in the regulation of large gene sets, thus fulfilling a novel molecular role at chromatin level.

## Supporting Information

Figure S1 **Nuclear colocalization of ERb and eNOS.** C27IM cells were cultured for 72 hours in hormone-deprived serum before treatment with E2 or the eNOS inhibitor L-NAME alone or in combination. Cells were stained with antibody to eNOS or ERβ and examined by confocal microscopy. Nuclei were stained with TOPRO3. Images were digitally transformed to quantify the mean fluorescence intensity on selected areas for single eNOS- or ERβ- positive cells. The resulting histograms indicate the presence or the accumulation of eNOS or ERβ proteins in the nuclei of prostate cells, expressed as fold induction relative to control (Nuclear localization index). Data represent the mean ± SEM of two indipendent experiments, each performed in duplicate. p<0,05 * vs control, # vs E2.
(TIF)

Figure S2 **Human chromosome ideograms of eNOS peaks in presence or absence of estradiol.** UCSC Genome Graphs illustrating the localization and density of eNOS-peaks in PCa cells: C27IM (A) and LNCaP (B); untreated (NT, blue dots) or treated with Estradiol (E2, red dots).
(TIF)

Figure S3 **Kernel Tag density analysis of eNOS-peaks distribution relative to the nearest TSS.** A) Window of 150.000 bp from TSS in C27IM_NT, C27IM_E2, LNCaP _NT, and LNCaP_E2 cells. B) Window of 10.000 bp from TSS: C27IM_NT versus C27IM_E2 (left) and LNCaP NT versus LNCaP E2 (right). Green dashed line: untreated samples (NT), red line: estradiol samples (E2). Welch Two Sample t-test of TSS distance C27IM E2 vs C27IM: p = 0.001166; LNCAP E2 vs LNCAP NT: p = 0.02002.
(TIF)

Figure S4 **Validation of ChIP-Seq eNOS peaks by MACS and SICER algorithms in C27IM and LNCaP cells, in the presence or absence of E2.** Peaks visualization was obtained using Integrated Genome Viewer (IGV 2.1). Screenshots of ChIP-Seq eNOS peaks surrounding hTERT (A), pS2 (TTF1, B) and GSTP1 (C) regulatory genomic regions are shown. Primers used for ChIP-qPCR validation are indicated with dashed orange lines.
(TIF)

Figure S5 A) Correlation heatmap using peak caller score between C27IM and LNCaP cells in the presence or absence of E2. B) Hierarchical cluster analysis of binding sites (affinity analysis) in C27IM and LNCaP cells with or without E2.
(TIF)

Figure S6 **Screenshot showing the pri-miR-34a genomic region and ChIP-Sequencing data.** Graphic representation of the alignement of reads derived from eNOS-immunoprecipitated and control input. MACS peaks in immunoprecipitated and control samples in C27IM untreated (NT) or treated with estradiol (E2) are shown in upper Panel, corresponding reads in lower Panel. eNOS-positive peaks corresponding to Figure 3B are indicated by red (C27IM_E2) and blue boxes (C27IM_NT). Transcription tracks from the ENCODE project: 1) overlayed H3K27Ac track shows where modification of histone proteins is suggestive of enhancer and, to a lesser extent, other regulatory elements; 2) DNase Clusters track shows regions where the chromatin is hypersensitive to DNase I, e.g regulatory regions and promoters); 3) Txn Factor ChIP track shows DNA regions where transcription factors bind as assessed by chromatin immunoprecipitation with antibodies specific to the transcription factor followed by sequencing of the precipitated DNA (ChIP-seq).
(TIF)

Table S1 ChIP-Seq mapping for each sample.
(DOC)

Table S2 Genome-wide determination of regions displaying eNOS-recruitment in the absence or presence of estradiol (MACS analysis).
(DOC)

Table S3 eNOS peak count per gene.
(DOC)

Methods S1 **Supplemental Methods for ChIP assays.**
(DOC)
