# Supplementary figures and images for: Estrogen-Dependent Dynamic Profile of eNOS-DNA Associations in Prostate Cancer
Source: PLoS One. 2013 May 3;8(5):e62522. doi: 10.1371/journal.pone.0062522 (PMC3643940; doi:10.1371/journal.pone.0062522)

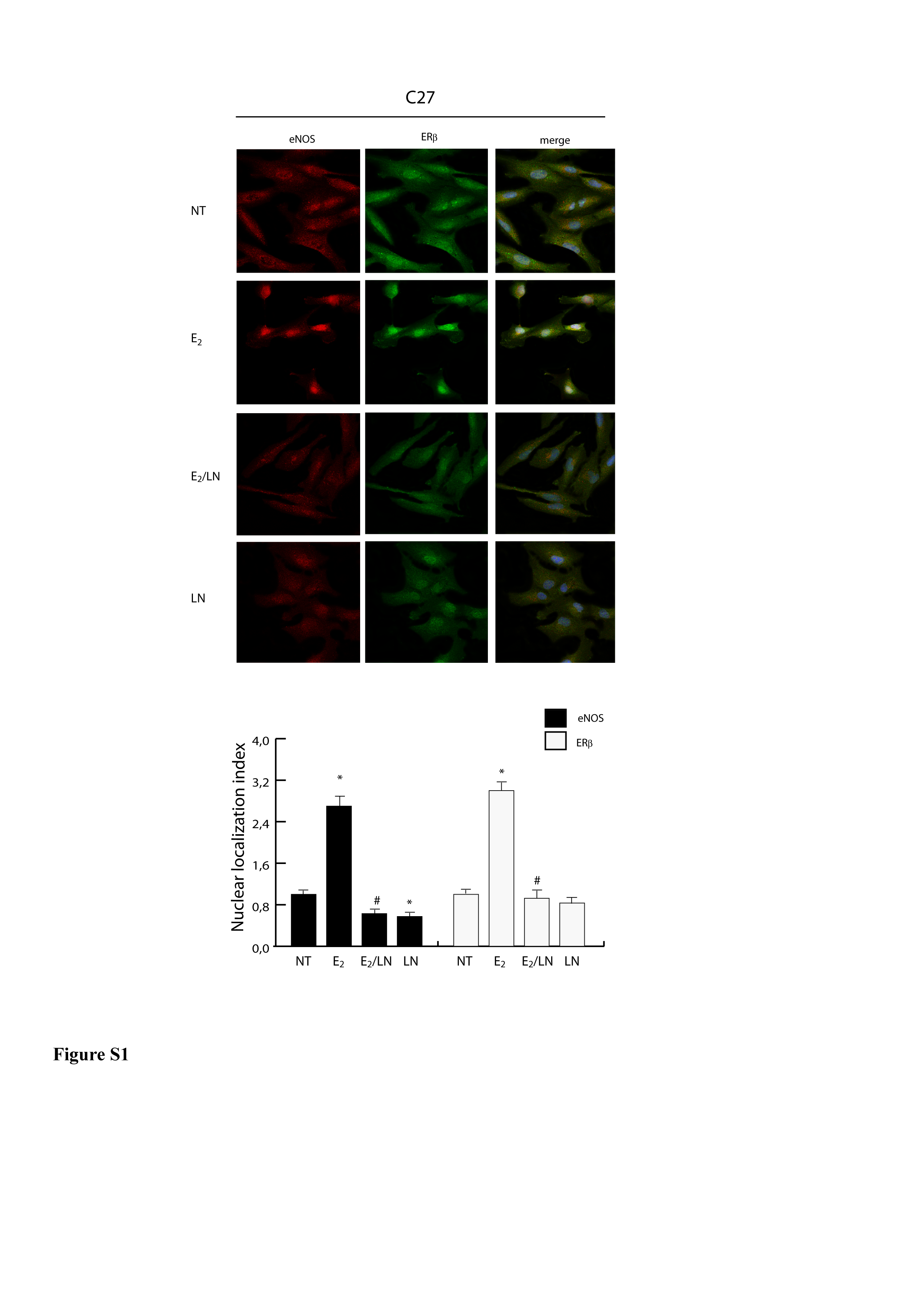

Supplement: Figure S1 — Nuclear colocalization of ERb and eNOS. C27IM cells were cultured for 72 hours in hormone-deprived serum before treatment with E2 or the eNOS inhibitor L-NAME alone or in combination. Cells were stained with antibody to eNOS or ERβ and examined by confocal microscopy. Nuclei were stained with TOPRO3. Images were digitally transformed to quantify the mean fluorescence intensity on selected areas for single eNOS- or ERβ- positive cells. The resulting histograms indicate the presence or the accumulation of eNOS or ERβ proteins in the nuclei of prostate cells, expressed as fold induction relative to control (Nuclear localization index). Data represent the mean ± SEM of two indipendent experiments, each performed in duplicate. p<0,05 * vs control, # vs E2. (TIF) [file pone.0062522.s001.tif]

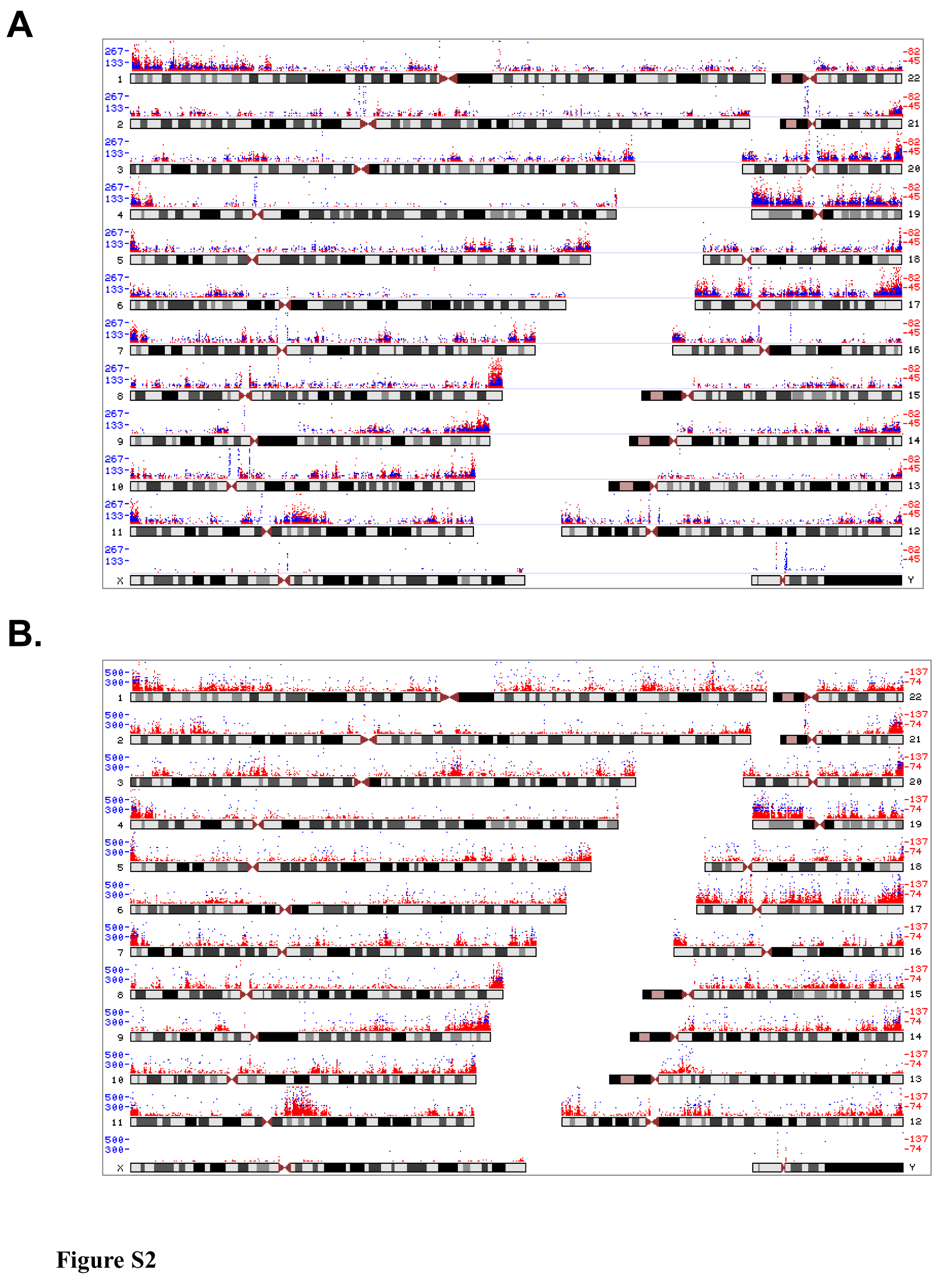

Supplement: Figure S2 — Human chromosome ideograms of eNOS peaks in presence or absence of estradiol. UCSC Genome Graphs illustrating the localization and density of eNOS-peaks in PCa cells: C27IM (A) and LNCaP (B); untreated (NT, blue dots) or treated with Estradiol (E2, red dots). (TIF) [file pone.0062522.s002.tif]

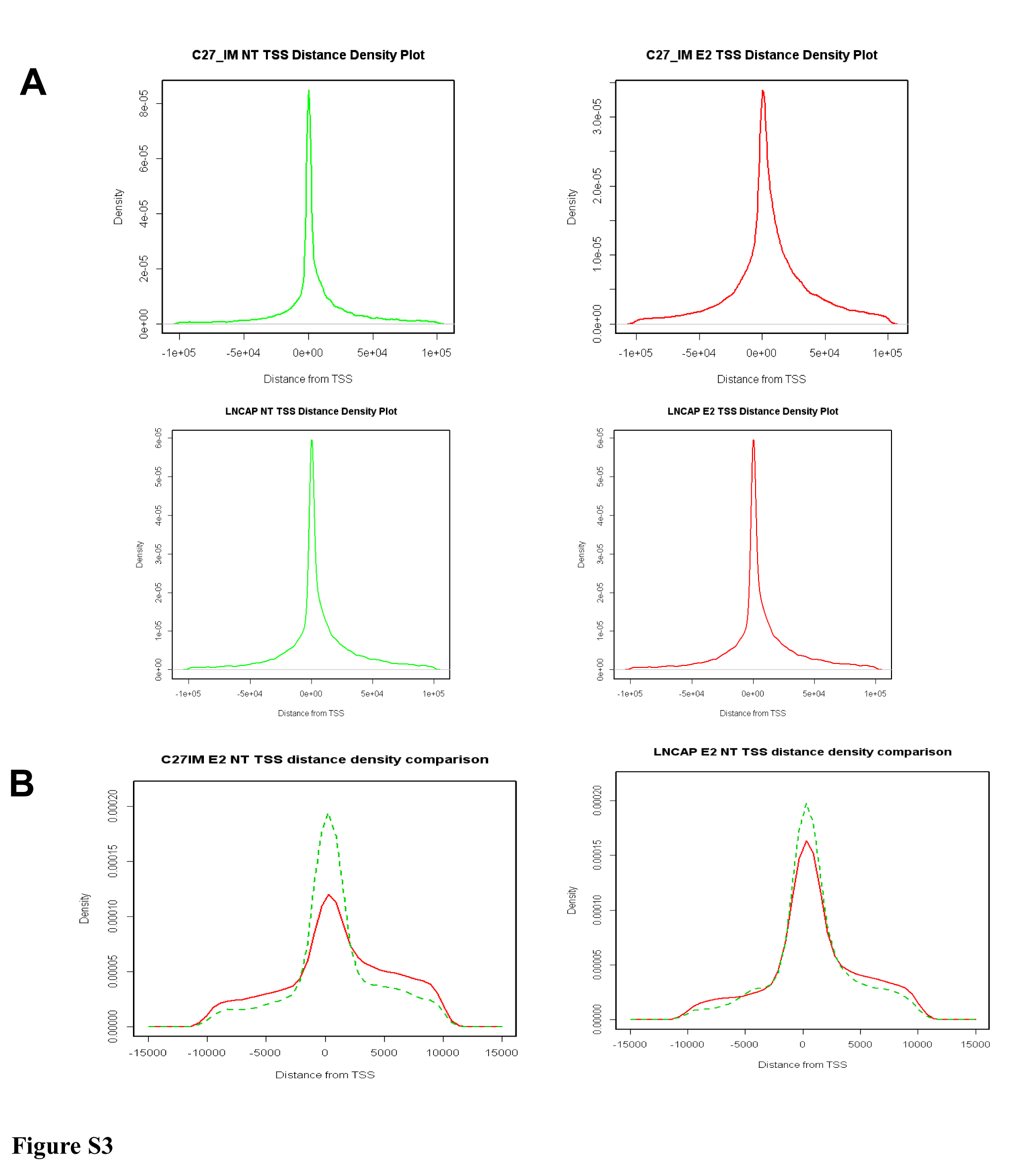

Supplement: Figure S3 — Kernel Tag density analysis of eNOS-peaks distribution relative to the nearest TSS. A) Window of 150.000 bp from TSS in C27IM_NT, C27IM_E2, LNCaP _NT, and LNCaP_E2 cells. B) Window of 10.000 bp from TSS: C27IM_NT versus C27IM_E2 (left) and LNCaP NT versus LNCaP E2 (right). Green dashed line: untreated samples (NT), red line: estradiol samples (E2). Welch Two Sample t-test of TSS distance C27IM E2 vs C27IM: p = 0.001166; LNCAP E2 vs LNCAP NT: p = 0.02002. (TIF) [file pone.0062522.s003.tif]

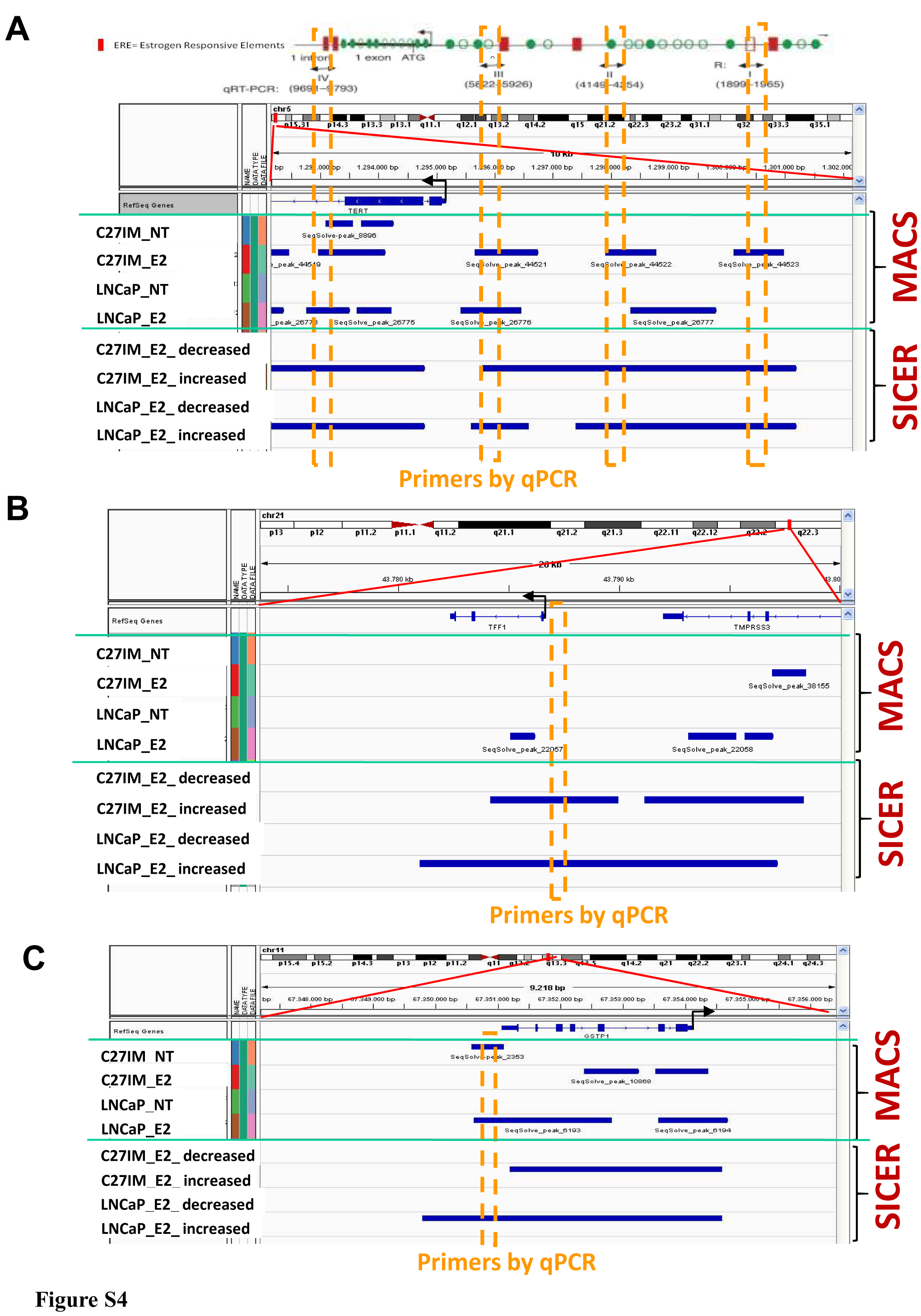

Supplement: Figure S4 — Validation of ChIP-Seq eNOS peaks by MACS and SICER algorithms in C27IM and LNCaP cells, in the presence or absence of E2. Peaks visualization was obtained using Integrated Genome Viewer (IGV 2.1). Screenshots of ChIP-Seq eNOS peaks surrounding hTERT (A), pS2 (TTF1, B) and GSTP1 (C) regulatory genomic regions are shown. Primers used for ChIP-qPCR validation are indicated with dashed orange lines. (TIF) [file pone.0062522.s004.tif]

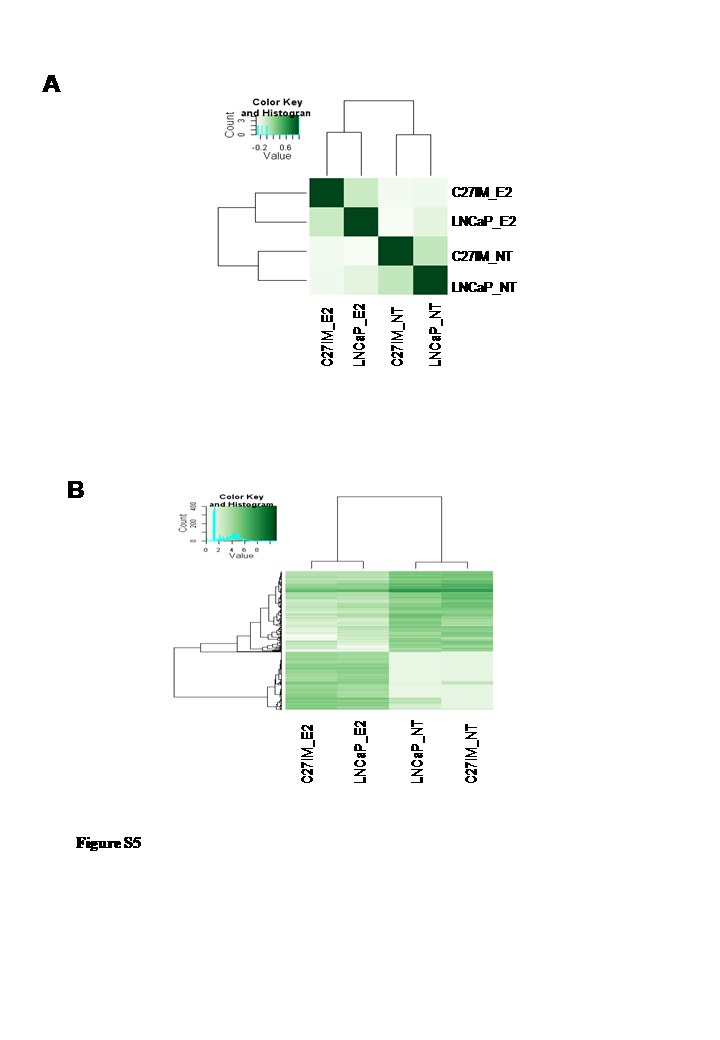

Supplement: Figure S5 — A) Correlation heatmap using peak caller score between C27IM and LNCaP cells in the presence or absence of E2. B) Hierarchical cluster analysis of binding sites (affinity analysis) in C27IM and LNCaP cells with or without E2. (TIF) [file pone.0062522.s005.tif]

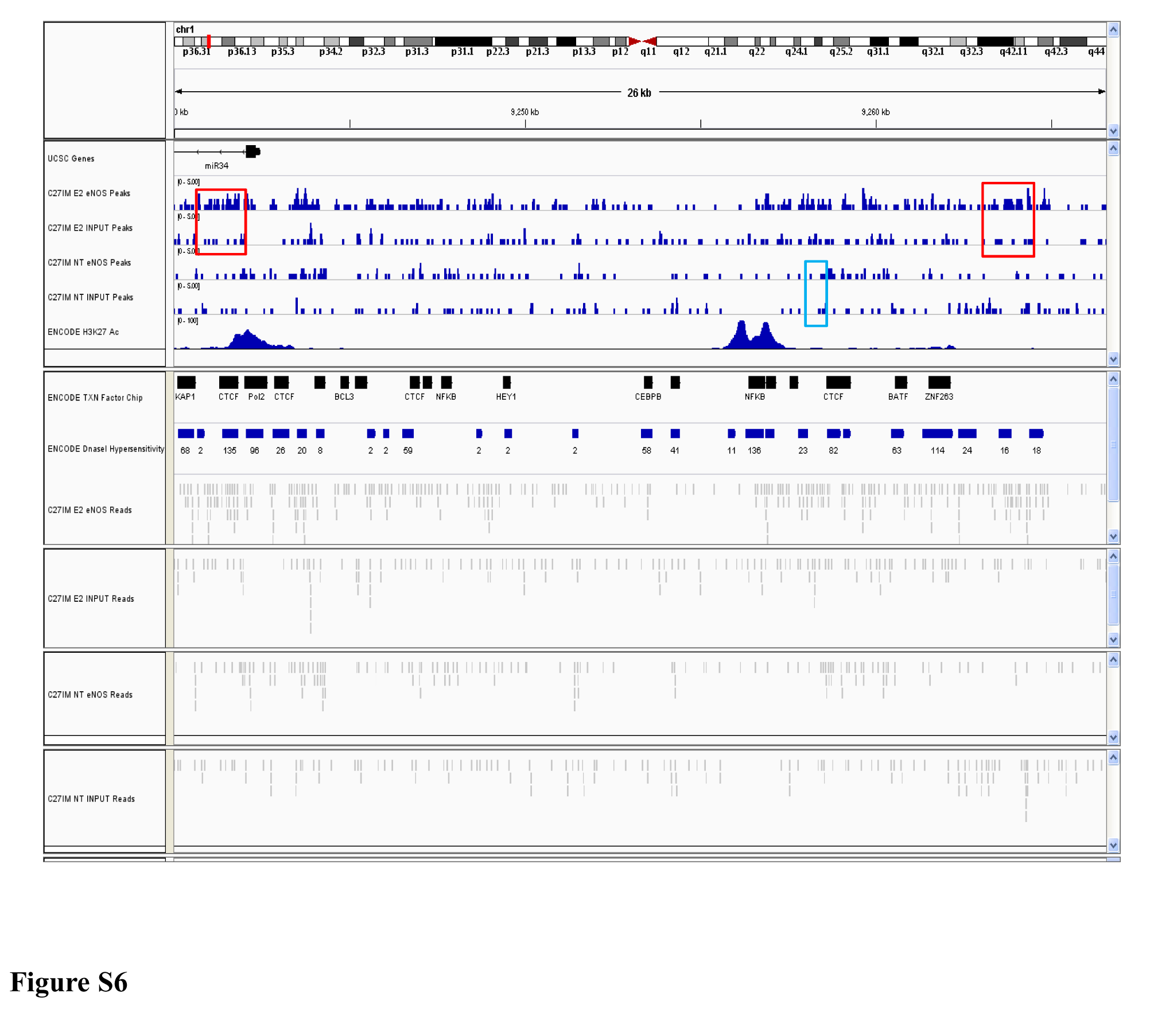

Supplement: Figure S6 — Screenshot showing the pri-miR-34a genomic region and ChIP-Sequencing data. Graphic representation of the alignement of reads derived from eNOS-immunoprecipitated and control input. MACS peaks in immunoprecipitated and control samples in C27IM untreated (NT) or treated with estradiol (E2) are shown in upper Panel, corresponding reads in lower Panel. eNOS-positive peaks corresponding to Figure 3B are indicated by red (C27IM_E2) and blue boxes (C27IM_NT). Transcription tracks from the ENCODE project: 1) overlayed H3K27Ac track shows where modification of histone proteins is suggestive of enhancer and, to a lesser extent, other regulatory elements; 2) DNase Clusters track shows regions where the chromatin is hypersensitive to DNase I, e.g regulatory regions and promoters); 3) Txn Factor ChIP track shows DNA regions where transcription factors bind as assessed by chromatin immunoprecipitation with antibodies specific to the transcription factor followed by sequencing of the precipitated DNA (ChIP-seq). (TIF) [file pone.0062522.s006.tif]
