# Supplementary material for: Estrogen-Dependent Dynamic Profile of eNOS-DNA Associations in Prostate Cancer
Source: PLoS One. 2013 May 3;8(5):e62522. doi: 10.1371/journal.pone.0062522 (PMC3643940; doi:10.1371/journal.pone.0062522)
Supplement: Table S1 — ChIP-Seq mapping for each sample. (DOC) [file pone.0062522.s007.doc]

**Table S1 . ChIP-Seq mapping for each sample.**

| **Samples** | **Sample type** | **Mapped reads number** |
| --- | --- | --- |
| C27IM_NT | ChIP | 9632735 |
| C27IM_NT | Input | 10698351 |
| C27IM_E2 | ChIP | 6950615 |
| C27IM_E2 | Input | 12486460 |
| LNCaP_NT | ChIP | 9187278 |
| LNCaP_NT | Input | 8405396 |
| LNCaP_E2 | ChIP | 7923462 |
| LNCaP_E2 | Input | 7732072 |
