# Supplementary material for: Estrogen-Dependent Dynamic Profile of eNOS-DNA Associations in Prostate Cancer
Source: PLoS One. 2013 May 3;8(5):e62522. doi: 10.1371/journal.pone.0062522 (PMC3643940; doi:10.1371/journal.pone.0062522)
Supplement: Table S2 — Genome-wide determination of regions displaying eNOS-recruitment in the absence or presence of estradiol (MACS analysis). (DOC) [file pone.0062522.s008.doc]

**Table S2. Genome-wide determination of regions displaying eNOS-recruitment in the absence or presence of estradiol (MACS analysis)**

| **Cells line** | **Treatment** | **Raw peak number ^a^** | **Depleted peak number ^b^** | **Peaks lenght, bp (Q1/Q3) ^c^** |
| --- | --- | --- | --- | --- |
| C27IM | Untreated (NT) | 12034 | 11694 | 596 (555/708) |
|  | Estradiol (E2) | 57802 | 57616 | 831 (624/1183) |
| LNCaP | Untreated (NT) | 2344 | 2333 | 596 (545/707) |
|  | Estradiol (E2) | 34560 | 34451 | 881 (666/1262) |

**^a^** number of discrete genomic regions in which eNOS-recruitment is observed (MACS analysis, FDR<0.1 and P value p<1e-05)

**^b^** number of peaks depleted of ultra-high signal artifact regions

**^c^** median size of peaks length, Q1=first quartile, Q3= third quartile of peaks-length distribution
