# Supplementary material for: Estrogen-Dependent Dynamic Profile of eNOS-DNA Associations in Prostate Cancer
Source: PLoS One. 2013 May 3;8(5):e62522. doi: 10.1371/journal.pone.0062522 (PMC3643940; doi:10.1371/journal.pone.0062522)
Supplement: Table S3 — eNOS peak count per gene. (DOC) [file pone.0062522.s009.doc]

**Table S3.** eNOS peak count per gene

| **Cells line** | **Treatment** | **1 peak (%)** | **2 peaks (%)** | **3 peaks (%)** | **4 or more peaks (%)** |
| --- | --- | --- | --- | --- | --- |
| C27IM | Untreated (NT) | 85% | 10.6% | 2.6% | 1.2% |
|  | Estradiol (E2) | 74% | 9.2% | 4.8% | 7.7% |
| LNCaP | Untreated (NT) | 95.8% | 3.6% | 0.3% | 0.3% |
|  | Estradiol (E2) | 72.5% | 11.6% | 5.3% | 10.4% |

Nanni et al. - Table 2 Supplemental– July 18th, 2012
