# Supplementary material for: Estrogen-Dependent Dynamic Profile of eNOS-DNA Associations in Prostate Cancer
Source: PLoS One. 2013 May 3;8(5):e62522. doi: 10.1371/journal.pone.0062522 (PMC3643940; doi:10.1371/journal.pone.0062522)
Supplement: Methods S1 — Supplemental Methods for ChIP assays. (DOC) [file pone.0062522.s010.doc]

**Supplemental Methods** **for ChIP assays:**

For ChIP assays the following primers were used:

hTERT promoter and pS2 promoter as in (3); GSTP1 promoter as in (5);

hSIRT1-promF 5’-TTGAACTTTTGACCTCGAGCAA-3’ and

hSIRT1-promR 5’-CAGCTGTAATCCCAACACTTTGG-3’;

hSIRT6promF 5’-AAACACACCCCCTCAAGATAGG-3’ and

hSIRT6promR 5’-CACCTGGGCCTGTTCTACGT-3’;

hKDRprom-F 5’-CGCCCGTTACCGAGTACTTTT-3’ and

hKDRprom-R 5’-AAGTCGCCCAGGAGAGAACA-3’;

miR-34a-prom-siteI-F 5’-CGCCTCCTTCTTTGCAAACT-3’ and

miR-34a-prom-siteI-R 5’-CACCCAGGTAAACGCTTGTGT-3’;

miR-34a-prom-siteII-F 5’-TCGGAGGGCCAATGCA-3’ and

miR-34a-prom-siteII-R 5’-GGTTCCTGGCTTTAGAAGTCCTT-3’;

miR-34a-prom-siteIII-F 5’-CTGGAAGGTGCAGGTCTTGTC-3’ and

miR-34a-prom-siteIII-R 5’-GACACCAGGAGCCTCTTGCT-3’;

miR-34a-prom-siteIV-F 5’-GGGACAGGAAAAAGCAACCA-3’ and

miR-34a-prom-siteIV-R 5’-TCGGTGACTGATGCCTCTCA-3’;

miR-34a-prom-siteV-F 5’-CCCAGTCCTTCCATTTCATGA-3’ and

miR-34a-prom-siteV-R 5’-CTAAGGCCCAGCCGTTTCT-3’;

miR-34a-prom-siteVI-F 5’-ACCGGGAAAAATACCAAGTTGTC-3’ and

miR-34a-prom-siteVI-R 5’-GACTTCAACCCTGTGCCTGTTC-3’;

h-miR143promF 5’-GCCCTGAGGCATGAAAAACA-3’ and

h-miR143promR 5’-TGCCGGAGTCCCTCTTTCT-3’;

h-let7b-promF 5’-GTGGAGTGGCACTTTTTCTTCTC-3’ and

h-let7b-promR 5’-TCCCCTCGACCTGCATGT-3’;

Chr5-F 5’-GCCATAGACTCCCAGGTTGAAG -3’ and

Chr5-R 5’-GTTGCACACCTGGTTCATTGA-3’.
